# Idiopathic verapamil-sensitive fascicular ventricular tachycardia with a potential direct connection of the antegrade limb to the retrograde myocardial limb, without bridging of the left posterior fascicle

**DOI:** 10.1016/j.hrcr.2024.12.001

**Published:** 2024-12-04

**Authors:** Kyong Hee Lee, Atsuhiko Yagishita, Iimura Kazuma, Mari Amino, Yuji Ikari, Koichiro Yoshioka

**Affiliations:** Department of Cardiology, Tokai University, Kanagawa, Japan

**Keywords:** Idiopathic fascicular ventricular tachycardia, Verapamil-sensitive, Posterior papillary muscle, Purkinje fibers, Catheter ablation

**Key Teaching Points**

• This case suggests a direct connection between the antegrade limb (P1) and retrograde myocardial conduction (V) without bridging of the left posterior fascicle (P2) in idiopathic verapamil-sensitive fascicular ventricular tachycardia (FVT) of the posterior papillary muscle subtype.
• The independence of the P2-V and P2-P2 interval prolongations during a radiofrequency application suggests that P2 acts as a bystander in the re-entrant circuit of the FVT.
• In conjunction with the alternation of the P1-V interval during FVT, this case not only supports the prevailing hypothesis that the left posterior fascicle acts as a bystander in the FVT circuit, but also indicates a connection within a confined area with potential multiple exits or an extensive bundle.

## Introduction

We present a case of idiopathic verapamil-sensitive fascicular ventricular tachycardia (FVT) of the posterior papillary muscle subtype. This case indicates a direct connection between the P1 potential (antegrade limb) and the myocardium without bridging to the P2 potential (left posterior fascicle), supporting the hypothesis that the left posterior fascicle acts as a bystander in the FVT circuit.

## Case report

A 17-year-old male patient presented with palpitations. A 12-lead electrocardiogram revealed regular wide QRS tachycardia (177 beats/min) with a right bundle branch block morphology and superior right axis. An echocardiogram revealed no structural abnormalities. Intravenous administration of verapamil (5 mg) successfully terminated the tachycardia.

**Figure 1:**
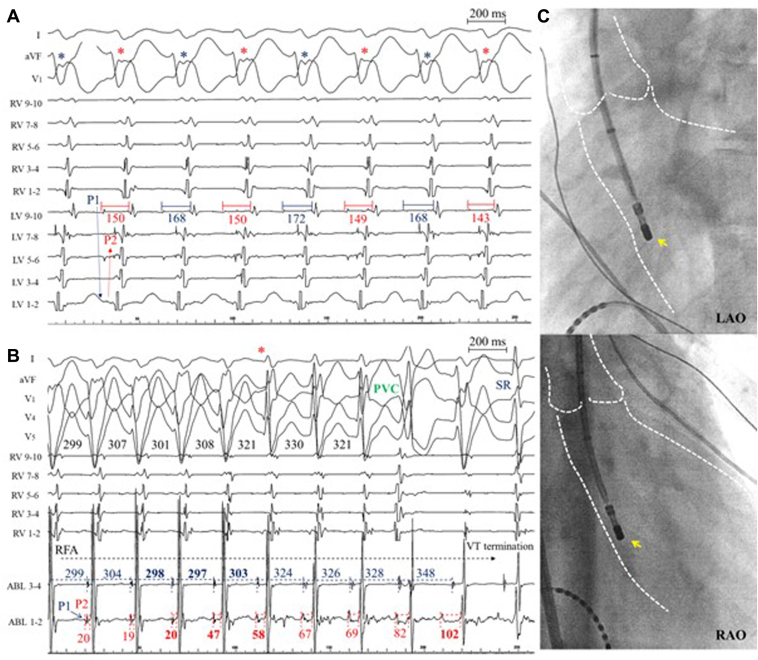
The alternation of tachycardia cycle length and QRS morphology and termination of the fascicular ventricular tachycardia during radiofrequency application. **A:** During left ventricular mapping, a mid-diastolic potential (P1) and an early presystolic potential (P2) were identified at the left ventricular septum. Note the alternation of the tachycardia cycle length and QRS morphology corresponding with changes in the P1-V interval. **B:** A radiofrequency application at the site where P1 and P2 potentials were recorded terminated the tachycardia. This termination was accompanied by changes in the axis from a superior right to left axis deviation. The dissociation was observed between prolongation of the P2-V interval with the P2-P2 interval. This suggests a direct connection between P1 and retrograde myocardial conduction without bridging of the P2 potentials. **C:** Fluoroscopic images in the left anterior oblique (LAO) view (*upper panel*) and right anterior oblique (RAO) view (*lower panel*) show the ablation catheter (ABL) (*yellow arrow*) positioned at the site in the left ventricle (LV) where P1 and P2 potentials were recorded, successfully terminating the tachycardia. PVC = premature ventricular contraction; RFA = radiofrequency application; RV = right ventricle; SR = sinus rhythm; V = ventricle; VT = ventricular tachycardia.

Catheter ablation was performed, and the clinical wide QRS tachycardia with a cycle length of 360 milliseconds was induced by means of ventricular programmed extra-stimuli. The atrioventricular dissociation, constant, and progressive fusion were observed. Left ventricular mapping with a 10-pole mapping catheter (DECANAV, Biosense Webster, Irvine, CA) revealed a mid-diastolic potential (P1) and an early presystolic potential (P2) at the left ventricular septum near the posterior papillary muscle. The P1 potential was recorded earlier on the proximal bipolar electrodes, and the P2 was recorded earlier on the distal bipolar electrodes. The tachycardia cycle length and QRS morphology alternated along with changes in the P1-V interval (Figure 1A). Radiofrequency application of 35 W using an ablation catheter (QDOT MICRO, Biosense Webster) at the site where P1 and P2 potentials were recorded terminated the tachycardia. This was accompanied by changes in the axis from a superior right to left axis deviation and prolongation of the P2-V interval (asterisk in Figure 1B) from 20 to 58 milliseconds (+38 milliseconds) within 3 consecutive beats, which was unrelated to the P2-P2 interval, changing from 298 to 303 milliseconds (+6 milliseconds). Following the continuous prolongation of the P2-V interval and, VT with a wider QRS morphology terminated with a prolonged P2-V interval of 102 milliseconds. This suggests a direct connection between the P1 potential and retrograde myocardial conduction without bridging of the P2 potential. During sinus rhythm, a delayed P1 potential was recorded in the distal bipoles of the ablation catheter. No VT induction or recurrence was observed during the 12 months of follow-up after catheter ablation.

## Discussion

**Figure 2:**
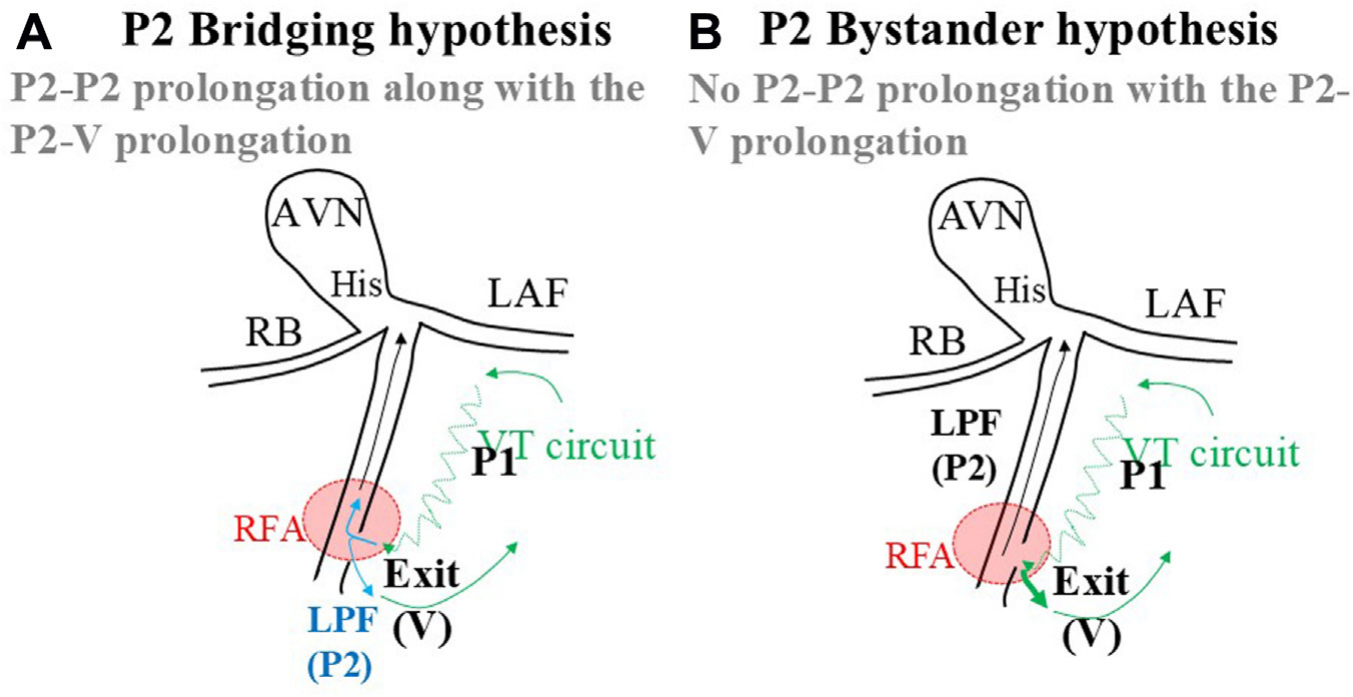
The postulated circuit of idiopathic verapamil-sensitive fascicular ventricular tachycardia of the posterior papillary muscle subtype. If the left posterior fascicle (P2) bridges the antegrade limb (P1) and the local papillary myocardium (V), the prolongation of the P2-P2 interval would be determined by the prolongation of the P2-V interval. **A:** P2 bridging hypothesis. Conversely, the independence of the P2-V and P2-P2 interval prolongations supports the notion of P2 as a bystander. **B:** P2 bystander hypothesis. AVN = atrioventricular node; LAF = left anterior fascicle; LPF = left posterior fascicle; RB = right bundle; RFA = radiofrequency application; VT = ventricular tachycardia.

This case raises the possibility of a direct connection between the antegrade limb and retrograde myocardial conduction, without the involvement of the left posterior fascicle, in idiopathic verapamil-sensitive FVT of the posterior papillary muscle subtype. This inference is based on observed differences in the prolongation of the P2-V and P2-P2 intervals before VT termination by RF application. If the left posterior fascicle were involved in connecting the antegrade limb and local papillary myocardium, the prolongation of the P2-P2 interval would align with that of the P2-V interval (Figure 2). The observed independence of these prolongations thus supports the hypothesis of P2 acting as a bystander, aligning with the idea that the left posterior fascicle may function as a bystander within the FVT circuit.^1^, ^2^, ^3^ In addition, this case is notable in its potential demonstration of a direct connection from the antegrade limb to the papillary myocardium, observed within the localized area where both P1 and P2 potentials were recorded on the distal bipoles of the ablation catheter. Furthermore, the alternating QRS morphologies and P1-V interval changes during FVT suggest the presence of multiple exits or an extensive bundle from the antegrade limb to the papillary myocardium.

## Conclusion

The present case suggests that the antegrade limb is directly connected to the myocardium without bridging to the left posterior fascicle in idiopathic verapamil-sensitive FVT of the posterior papillary muscle subtype. This case not only supports the prevailing hypothesis that the left posterior fascicle acts as a bystander in the FVT circuit, but also indicates a direct connection between antegrade and retrograde myocardium within a confined area with potential multiple exits or an extensive bundle.

## Disclosures

The authors have no conflicts of interest to disclose.
